# Notes and Notices

**Published:** 1867-04

**Authors:** 


### NOTES AND NOTICES.

SPRING WATER.—The waters of the Excelsior Spring, at Saratoga, may be had at 47 Warren street, New York, at a moderate cost from the agent, Mr. William D. Forster, whose courtesy to all who call upon him personally, and whose promptitude and fidelity in the fulfilment of orders from abroad, are well known. Dr. Oliver Wendell Holmes says, "I find it possessing in a marked degree, all the medicinal qualities mentioned by Professor Smith, of Baltimore, and Professor Dickson, and exceedingly gentle and uniform in its effects." Dr. Casper Morris says, emphatically, that "it possesses all the merits of all the other Saratoga waters." It is tonic, alterative, diuretic and cathartic, according to the proportions used.

"Cerebro Spinal Meningitis," being a report made to the Illinois State Medical Society, at Decatur, by J. S. Jewell, M. D., Professor of Anatomy, Chicago Medical College. This is by far the most complete, full and scientific paper we have yet seen on the malady in question, and every educated physician who has not seen it, should send at once for this valuable monogram, to George H. Fergus, 14 Clark street, Chicago, Illinois; sent, post-paid, for 25c.

*Feeble Minded Children* are educated at Barre, Mass. George Brown, M. D., Superintendent, to whom persons interested can write for a report on the condition, prospects and success of this admirable Institution.

The American Tract Society, 150 Nassau street, New York, have published "The Day Dawn," by the author of "Memorials of Capt. Hedley Vicars," and "English Hearts and English Hands," also "Thy Day," a word to all, by S.M. Haughton, author of "A Saviour for You," &c., &c., "Faith," What it is, and What it Does, by same. "The Reign of Grace," by Thomas Chalmers, D. D. Also a catalogue of volumes and libraries, adapted to the use of Pastors, Families, Bible Classes and Sabbath Schools, this last sent, post-paid, for six cent stamp, the other four, all sent by mail, post-paid, for 25c.

"The Story of a Stomach," an Egotism, by a Reformed Dyspeptic, published and sent, post-paid, for 30c. by Fowler & Wells, 389 Broadway, New York. Also for 25c.

"Thoughts on Domestic Life," or Marriage Vindicated and Free Love Exposed, by Nelson Sizer. "Let every man have his own wife, and let every woman have her own husband," 1 Corinthians, 7 : 2.

"Temperance." Rev. James B. Dunn has prepared a Temperance Catechism, for the use of temperance societies and private persons, containing a great deal of very interesting, striking and useful information. 6c. Address, J. N. Stearns, 172 William street, New York.

"Unity," and its restoration, addressed to all Christians, by a Presbyter of the Diocese of Illinois, dedicated to C. B. T., "greatly beloved," by whose sick bed, in the night watches of sanctified pain, the pages were written, in the cause of the unity of that church, in whose communion he died." We do not believe in the sentiments of the author of this pamphlet; like every other writer we have seen on this subject, our author makes the chief stone of the corner, his own infallibility, which expresses itself thus, "if all Christians every where believe as I do, there will be a good time coming soon." A one sided church like a one sided government, where there is but one political party, would become corrupt and be disorganized; it is the friction of thought with thought, that brings out the sparkles of truth in sentiment and practice. The Christian Church in its present organization, numerous like the billows, but as the ocean one, has made a good fight thus far, under its different banners, and no doubt will continue to do so, until its influence will "cover the earth, as the waters do the face of the great deep."

The Messrs. Broughton & Tuyman, 13 Bible House, New York, and 28 Cornhill, Boston, have sent us three pretty little books for children, "Lucy and Bell," and how they over came, 35c., "Christmas at the Beeches," 50c., and "The Blue Book Stories," by Harriet F. Woods, 203 pages, 80c.; all calculated to entertain the mind, instruct the head, and improve the heart. All sent, post-paid, for prices named.

"The Beacon," by Dr. Wm. M. Cornell, No. 7 Maynard Place, Boston, Mass., a conscientious physician and well known writer, has published a book with this title, which will be read with peculiar interest by those who are troubled with erysipelas and other ailments affecting the mind; it is sent, post-paid, for 50c. and may be profitably read by old and young. He has also written a practical work of great value, entitled, "How to enjoy life, or physical and mental Hygiene." The very large number of commendations bestowed on this publication by the press, without any known exception, are indications of the real value of the book.

"Phillip the II of Spain," by Charles Gayerre, author of the "History of Louisiana," with an introductory letter by George Bancroft, is just published by W. J. Widdleton, 17 Mercer street, New York. Mr. B. says that this book, "like that of Mr. Rives, has an important connection with the great questions which the mind of America is now engaged in solving. Both of them are indirectly the highest tributes to the incomparable excellency of our institutions, and are the most earnest admonitions in favor of their perpetuity." This valuable work is in a measure the continuation of Prescott's unfinished history of this stern son of Charles the Fifth, styled, "The Demon of the South."

"Conservative Surgery," as exhibited in surveying some of the mechanical causes which operate injuriously both in health and disease, with illustrations, by Henry G. Davis, Member of the American Medical Association, &c. &c., is published by D. Appleton & Co., 445 Broadway, New York. These records are a present to the Medical Profession of the gradual accumulations of over thirty years investigation. This is an endeavor to discover and elucidate principles, and is the only method of making true progress, and the work is confidently commended to the notice of practitioners in medicine and surgery. The subjects treated are fractures, dislocations, joints, muscles, tendons, deformities, spina! curvatures, symvites, morbus ceravius, joint diseases, &c., &c.

"Gay Cottage," by Glance Gaylord, a beautiful little vol. of 144 pages of the American Tract Society 13 Bible House, New York, being a beautiful story beginning with "Going after a City Boy;" also "Steps in the Upward Way" or the Story of Fanny Bell by Mary Barrett, author of "Shooting at a Mark," etc. It contains 279 pages of profitable reading matter, to old and young, for parents and children, giving some boarding school revelations, from the interior—The first deception—The Telegram—Homeward—On Jordan's strand—&c., 24 chapters in all, and all very interesting. Any of our readers who will send their orders to Messrs. Broughton & Tuyman No. 13 Bible House, New York, will have them promptly and justly attended to.

